# Predicting 5-Year Mortality in Non–Small-Cell Lung Cancer Using the Korean Central Cancer Registry: Model Development and Validation Study

**DOI:** 10.2196/80574

**Published:** 2026-06-08

**Authors:** Jong Hyuk Lee, Ho Cheol Kim, Kyu-Won Jung, Chang Min Choi

**Affiliations:** 1 Department of Oncology, College of Medicine, Asan Medical Center, University of Ulsan, Seoul, Republic of Korea; 2 Department of Pulmonary and Critical Care Medicine, College of Medicine, Asan Medical Center, University of Ulsan, 88, Olympic-ro 43-gil, Songpa-gu, Seoul, 05505, Republic of Korea, +82-10-9792-9607; 3 Division of Cancer Registration and Surveillance, National Cancer Control Institute, National Cancer Center, Goyang, Republic of Korea

**Keywords:** non–small-cell lung cancer, deep learning, machine learning, prognosis, Korean Central Cancer Registry, KCCR, permutation tests

## Abstract

**Background:**

Non–small-cell lung cancer (NSCLC) is one of the most common cancers and a leading cause of cancer-related mortality, making prognostic prediction clinically essential. Machine learning models are increasingly used to assess prognosis; however, developing systems that combine high discrimination with clear, clinically interpretable reasoning remains challenging.

**Objective:**

This study aimed to develop deep learning models that predict 5-year mortality in NSCLC using data from the Korea Central Cancer Registry and quantify feature importance through permutation testing.

**Methods:**

We identified 3144 patients diagnosed between 2014 and 2017 who had complete clinical data, pulmonary function test results, histological information, genomic data, and staging details. After preprocessing, the cohort was divided into stratified training, validation, and test sets in a 70%-15%-15% ratio. Five models were tuned using Hyperband across 10 predefined feature groups. The primary evaluation metric was the area under the receiver operating characteristic curve (AUC); additional metrics included accuracy, *F*_1_-score, precision, and recall. Groupwise permutation importance was calculated for each model, and the concordance of importance rankings was assessed using the Friedman test.

**Results:**

All 5 models yielded comparable discrimination values on the test set (AUC=0.875‐0.879). Model A was selected as the primary model and achieved an AUC of 0.879, an accuracy of 0.806, an *F*_1_-score of 0.824, and a Brier score of 0.142. Permuting the stage resulted in the largest decrease in AUC (0.217), followed by the pulmonary function test (0.016). Gene mutation had a modest overall impact but became more influential within the adenocarcinoma subset. The Friedman test showed no statistically significant differences in importance rankings across the models (*P*=.93).

**Conclusions:**

A grouped-input deep learning framework achieved discrimination comparable to a conventional Cox proportional hazards model using the same routine clinical variables for 5-year mortality prediction in NSCLC. Group-level permutation importance provided stable and reproducible insights into the clinical factors influencing risk, which may guide future model refinement and clinical decision-making.

## Introduction

Non–small-cell lung cancer (NSCLC) is one of the most prevalent cancers worldwide and, despite advancements in prognosis, remains a leading cause of cancer-related mortality [1-3]. The prognosis of NSCLC is influenced by various factors, including the stage at diagnosis and molecular biomarkers such as alterations in the epidermal growth factor receptor (EGFR) and anaplastic lymphoma kinase (ALK). Therefore, predicting an individual patient’s clinical course is crucial for tailoring treatment strategies [2,4,5].

Machine learning has gained significant traction in oncology due to its ability to capture complex, nonlinear relationships and integrate heterogeneous data modalities [6,7]. Among the various approaches, deep learning stands out for its strong predictive performance and capacity to automatically learn features [8]. However, its limited interpretability and reliance on large, well-curated datasets hinder broad adoption in medical research [9,10]. Despite these challenges, many researchers continue to explore deep learning solutions, and growing evidence indicates that this approach enhances decision-making and supports tasks ranging from screening and diagnosis to prognostic prediction in lung cancer and other malignancies [11-14]. Nevertheless, many previous studies have relied on small, single-institution cohorts, increasing the risk of overfitting, and often inadequately reported data preprocessing or hyperparameter tuning procedures, thereby limiting reproducibility [5,11]. Assembling a sufficiently large, well-curated dataset and developing a clinically practical model remain significant challenges for clinicians.

The Korea Central Cancer Registry (KCCR) is a national population-based registry that systematically collects data on all patients with cancer diagnosed in South Korea. For lung cancer, the KCCR enhanced the staging survey, which samples 10% of all incident lung cancer cases each year. This survey includes demographic variables and clinical details such as symptoms; tumor, node, metastasis (TNM) stage; histologic subtype; and biomarker profiles [15]. By aggregating deidentified records from over 50 medical centers, the KCCR offers a large, multicenter cohort suitable for robust deep learning analyses. This dataset is particularly suitable for deep learning analyses because it captures complex interactions among heterogeneous clinical variables.

Accordingly, we developed and validated a deep learning model for 5-year mortality prediction in NSCLC using nationwide registry data from the KCCR and routinely collected clinical features. We implemented a grouped-input architecture that mirrors clinically coherent feature domains and incorporated permutation-based, group-level importance to provide a transparent and reproducible assessment of feature contributions across model configurations [16]. Collectively, this design targets clinically applicable use with limited routine variables while maintaining interpretability and clear reporting of preprocessing and hyperparameter tuning.

## Methods

### Data Selection, Preprocessing, and Stratified Splitting

We obtained data from the KCCR nationwide lung cancer staging survey, which includes patients with NSCLC from 52 hospitals across South Korea. Records from patients diagnosed between 2014 and 2017 were used, excluding those with missing or uncertain information regarding TNM stage, gene mutations (EGFR or ALK), or diagnosis date. Patients with logically inconsistent smoking cessation dates (eg, a missing cessation date despite being recorded as a former smoker, a cessation date earlier than the smoking start date, or a cessation date later than the date of death) were also excluded. An Eastern Cooperative Oncology Group (ECOG) performance status recorded as “unknown” was recoded as 0. For records with “unknown” smoking history, the smoking amount and duration fields were also missing; therefore, these cases were recoded as never smokers. We used a complete-case approach for the core variables to avoid unverifiable imputation assumptions and ensure consistency across clinical feature domains. Because this approach may introduce selection bias, we compared baseline characteristics between included and excluded patients using absolute standardized mean differences (SMDs).

A comprehensive set of demographic and clinical variables was extracted from the original records, including age, sex, BMI (calculated from weight and height), smoking history, symptoms, ECOG performance status, pulmonary function test (PFT) results, clinical stage, and pathological features. For PFT variables, we included one-hot availability indicators derived from the PFT status field along with scaled continuous measurements. Unmeasured values (ie, when the test was not performed) were coded using a sentinel value to indicate nonavailability rather than physiologic zero. Smoking status was one-hot encoded, and quit duration was modeled as a continuous variable only for former smokers. We used a sentinel value for never smokers to denote “not applicable,” whereas quit duration was set to 0 for current smokers. To assess the potential impact of recoding missing clinical information to the healthiest baseline, we additionally trained an “unknown-aware” version of our primary model in which “unknown” ECOG performance status and smoking history were retained as distinct categories. The same data splits, model architecture, and evaluation procedures were used for direct comparison.

Five-year mortality and survival were defined as an interval of at least 60 months between diagnosis and death or last follow-up. Patients who met this threshold were classified as 5-year survivors, whereas the others were categorized as nonsurvivors. Detailed preprocessing steps are summarized in Table S1 in Multimedia Appendix 1.

The resulting data were stratified by sex, age, smoking history, PFT status (performed or not), gene mutation status, and 5-year mortality. Accordingly, 70% of patients were allocated to the training dataset, 15% were allocated to the validation set, and 15% were allocated to the test set. Immediately before model training, all continuous variables were scaled to the range of 0 to 1 using min-max normalization, with parameters estimated solely from the training subset. The same transformation was then applied unchanged to the validation and test subsets. Categorical variables were one-hot encoded in a similar manner: the encoder was fitted on the training set and reused for the other sets. Symptoms and histology were recorded as binary indicators (0=absent; 1=present). Instead of encoding each variable separately, we combined the symptom and histology indicators into a single composite vector and applied one-hot encoding. EGFR and ALK mutation indicators were handled similarly, being merged into 1 vector prior to one-hot encoding.

### Model Construction and Hyperparameter Tuning

**Figure 1.**
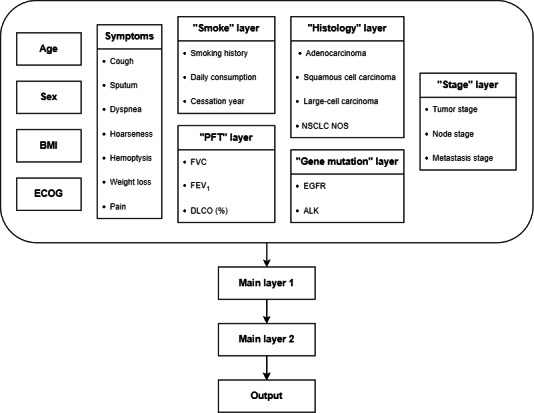
Grouped-input multilayer perceptron architecture for 5-year mortality prediction in non–small-cell lung cancer (NSCLC). Input variables were organized into clinically defined feature domains (demographics; symptoms; smoking variables; pulmonary function test [PFT] measurements; histology; gene mutation; and tumor, node, metastasis stage), processed through domain-specific input layers, and merged into shared hidden layers to generate the final prediction. ALK: anaplastic lymphoma kinase; DLCO: diffusion capacity of the lungs for carbon monoxide; ECOG: Eastern Cooperative Oncology Group performance status; EGFR: epidermal growth factor receptor; FEV_1_: forced expiratory volume in 1 second; FVC: forced vital capacity; NOS: not otherwise specified.

A multilayer perceptron (MLP) was developed to predict 5-year mortality (Figure 1). The model consists of 2 primary layers preceding the output layer. Age, sex, BMI, ECOG score, and symptoms were directly input into the first main layer, whereas other features (eg, smoking status, PFT, stage, histology, and gene mutation) were grouped into dedicated sublayers. This grouped-input design mirrors clinically coherent feature domains, improving interpretability and enabling domain-level regularization (eg, group-specific widths and dropout) in the presence of correlated predictors; it also stabilizes training under sparsity and collinearity. The outputs from these sublayers were then concatenated and fed into the first primary layer. All sublayers and primary layers used rectified linear unit activation functions, whereas the output layer used a sigmoid function for binary classification. Dropout and L2 regularization techniques were applied throughout these layers to reduce the risk of overfitting.

Our model was implemented in Python (version 3.12.5; Python Software Foundation) using TensorFlow (version 2.18; Google Brain Team) and scikit-learn (version 1.6.1; Google Summer of Code project). We used the Adam optimizer and binary cross-entropy loss for training. Hyperparameters were tuned using the Keras Hyperband tuner. We selected Hyperband because it efficiently explores large search spaces by allocating more epochs to promising configurations and aggressively pruning unpromising ones (successive halving with early stopping), thereby reducing computational cost and overfitting risk. The hyperparameters were evaluated across the following ranges: sublayer units (4, 8, or 16 for most features and 8, 16, or 32 for stage), main layer units (32, 64, or 128), dropout rates (0.30‐0.50 in increments of 0.05), L2 regularization strength (λ in 10^–6^ to 10^–3^; log scale), and learning rate (10^–4^ to 10^–2^; log scale).

Each candidate architecture was trained for up to 100 epochs, with successive Hyperband rounds retaining approximately the top one-third of models and granting them progressively larger training budgets. The optimization target was the area under the receiver operating characteristic curve (AUC), evaluated on the held-out validation cohort. During each trial, an early-stopping callback monitored the same metric and restored the best weights after 5 epochs without improvement. This approach helped prevent overfitting and reduced runtime. The batch size was fixed at 32. This configuration facilitated efficient evaluation of numerous hyperparameter combinations, allowing for the identification of a set that delivered robust performance with minimal overfitting.

### Performance Evaluation

AUC was prespecified as the primary metric given outcome imbalance and its threshold-free nature; accuracy, precision, recall, *F*_1_-score, and the area under the precision-recall curve (AUPRC) were reported for completeness. Each metric was calculated for the training, validation, and test datasets. We estimated the 95% CIs through bootstrapping with 1000 resamples. Because our primary objective was to determine whether a patient survived for 5 years, AUC was regarded as the main criterion for performance evaluation and for detecting overfitting. The target for the test set was an AUC exceeding 0.800, with the difference in AUC between the training and test sets remaining below 0.05.

Cox proportional hazards (CPH) models were established as baselines for comparison using the same predictors. First, a full CPH model was fitted using the same set of predictors as the MLP models. In addition, a TNM-only CPH benchmark model was constructed using only the tumor, node, and metastasis variables to quantify the incremental predictive value of nonstaging clinical features beyond TNM staging. Predicted 5-year mortality probabilities were evaluated using the same set of discrimination and classification metrics across models, enabling direct performance comparisons between the CPH and MLP models.

### Permutation-Based Feature Importance

**Figure 2.**
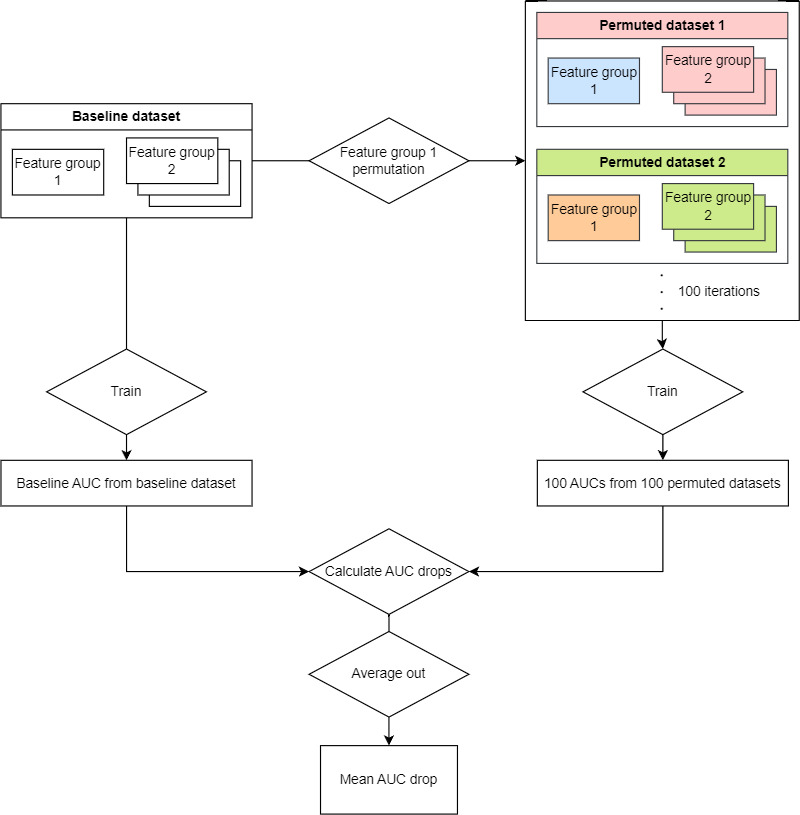
Schematic overview of the grouped-feature permutation importance procedure. For each clinically defined feature group, values were permuted, whereas the features in all other groups were held fixed, generating 100 permuted datasets. The trained model was evaluated on each dataset, and feature group importance was quantified as the decrease in area under the receiver operating characteristic curve (AUC) relative to the baseline (mean AUC drop across iterations).

A permutation-based approach was used to assess the importance of each feature (Figure 2). First, a baseline model was trained using the original test dataset to establish a reference AUC. Then, for each individual feature or feature group, we randomly permuted its variables 100 times while keeping the other features intact and retrained the model on each permuted dataset. The resulting AUC values were compared with the baseline AUC, and the average reductions across permutations were calculated to obtain a mean reduction. A greater reduction indicates a higher contribution of that feature group to the predictive performance of the model.

The Friedman test using R (version 4.4.0; R Foundation for Statistical Computing) was conducted on the mean AUC reductions to assess consistency across multiple model configurations. This statistical test evaluates the rank order of feature group importance among models to determine whether significant differences in feature rankings exist. A *P* value below .05 was considered statistically significant.

### Ethical Considerations

The institutional review board of Asan Medical Center approved this retrospective study (2025-0572; approval date: May 13, 2025). The requirement for written informed consent was waived by the institutional review board because this study involved secondary analysis of routinely collected registry data and posed minimal risk to participants. The investigators received a deidentified dataset from the KCCR and had no access to directly identifiable personal information. Data were analyzed in accordance with institutional policies on data security and confidentiality. No compensation was provided, as participants were not prospectively recruited and there was no direct contact with patients. The manuscript and supplementary materials do not include any images or information that could identify individual participants.

## Results

### Baseline Demographics of the Patients

**Figure 3.**
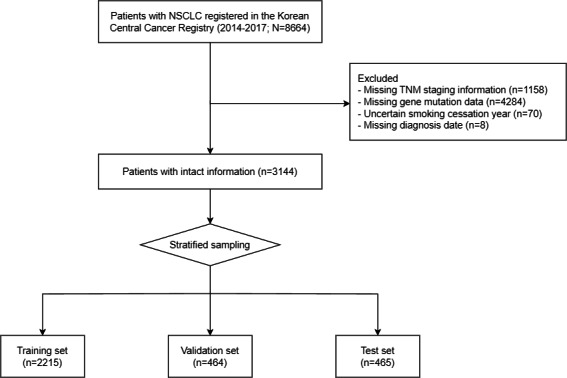
Flowchart of cohort selection and dataset splitting for a retrospective registry-based study of non–small-cell lung cancer (NSCLC) in South Korea using the Korea Central Cancer Registry (2014‐2017). Of 8664 registered patients, 5520 (63.7%) were excluded due to missing tumor, node, metastasis (TNM) stage information; missing gene mutation data; uncertain smoking cessation year; or missing diagnosis date. The final analytic cohort included 3144 patients and was split using stratified sampling into training, validation, and test sets.

A total of 8664 patients diagnosed with NSCLC from 2014 to 2017 were identified in the KCCR. Of these 8664 patients, 3144 (36.3%) had complete data suitable for analysis. Stratified sampling was conducted to assign patients to the training (2215/3144, 70.5%), validation (464/3144, 14.8%), or test (465/3144, 14.8%; Figure 3) groups. Additionally, 50 patients who lacked matching counterparts during stratification were included in the training set.

Patients’ baseline characteristics are summarized in Table 1. Among the total cohort, 60.7% (1908/3144) of the participants were male. The median age at diagnosis was 66 (IQR 58‐74) years. Most patients had an ECOG performance status of 0 (1902/3144, 60.5%), followed by a score of 1 (1023/3144, 32.5%). Table 2 presents details of the cancer-related characteristics. Most patients (2490/3144, 79.2%) were diagnosed with adenocarcinoma. Regarding stage, 35.4% (1113/3144) of the patients were diagnosed at stage 1, whereas the largest subgroup was those diagnosed at stage 4 (1282/3144, 40.8%). EGFR mutations were detected in 36.0% (1131/3144) of the patients, whereas ALK mutations were less common, occurring in 6.6% (209/3144) of the patients. A total of 42.9% (1350/3144) of the patients survived at least 5 years after diagnosis. There were no statistically significant differences in baseline characteristics among the training, validation, and test cohorts.

**Table 1.** Baseline demographic and clinical characteristics of the included patients (N=3144).

	Overall	Training set (n=2215)	Validation set (n=464)	Test set (n=465)	*P* value
Sex, n (%)					.89
Male	1908 (60.7)	1340 (60.5)	281 (60.6)	287 (61.7)	
Female	1236 (39.3)	875 (39.5)	183 (39.4)	178 (38.3)	
Age (y), median (IQR)	66 (58‐74)	66 (58‐74)	66 (58‐74)	66 (58‐74)	.89
BMI (kg/m^2^), median (IQR)	23.2 (21.0‐25.4)	23.3 (21.0‐25.4)	23.0 (21.1‐25.4)	23.0 (20.8‐25.4)	.96
ECOG^a^ performance status, n (%)					.77
0	1902 (60.5)	1330 (60.0)	282 (60.8)	290 (62.4)	
1	1023 (32.5)	726 (32.8)	150 (32.3)	147 (31.6)	
2	160 (5.1)	114 (5.1)	23 (5.0)	23 (4.9)	
3	45 (1.4)	32 (1.4)	8 (1.7)	5 (1.1)	
4	14 (0.4)	13 (0.6)	1 (0.2)	0 (0)	
Smoking status, n (%)					>.99
Never smoked	1420 (45.2)	1000 (45.1)	210 (45.3)	210 (45.2)	
Current smoker	927 (29.5)	654 (29.5)	136 (29.3)	137 (29.5)	
Ex-smoker	797 (25.3)	561 (25.3)	118 (25.4)	118 (25.4)	
PFT^b^, median (IQR)					
FVC^c^ (L)	3.1 (2.6‐3.7)	3.1 (2.6‐3.7)	3.1 (2.6‐3.8)	3.1 (2.5‐3.7)	.76
FEV_1_^d^ (L)	2.3 (1.8‐2.7)	2.3 (1.8‐2.7)	2.3 (1.9‐2.8)	2.2 (1.8‐2.7)	.66
DLCO^e^ (%)	85.0 (71.0‐99.0)	85.0 (71.0‐99.0)	86.5 (72.0‐100.0)	83.0 (71.0‐97.0)	.58

a ECOG: Eastern Cooperative Oncology Group.

b PFT: pulmonary function test.

c FVC: forced vital capacity.

d FEV_1_: forced expiratory volume in 1 second.

e DLCO: diffusion capacity of the lungs for carbon monoxide.

**Table 2.** Cancer-related characteristics of the included patients (N=3144).

	Overall, n (%)	Training set (n=2215), n (%)	Validation set (n=464), n (%)	Test set (n=465), n (%)	*P* value
Symptoms					>.99
Asymptomatic	1528 (48.6)	1067 (48.2)	235 (50.6)	226 (48.6)	
Cough	890 (28.3)	624 (28.2)	138 (29.7)	128 (27.5)	
Sputum	494 (15.7)	345 (15.6)	76 (16.4)	73 (15.7)	
Dyspnea	431 (13.7)	301 (13.6)	62 (13.4)	68 (14.6)	
Hoarseness	34 (1.1)	22 (1.0)	7 (1.5)	5 (1.1)	
Hemoptysis	147 (4.7)	102 (4.6)	21 (4.5)	24 (5.2)	
Weight loss	185 (5.9)	133 (6.0)	27 (5.8)	25 (5.4)	
Pain	493 (15.7)	352 (15.9)	77 (16.6)	66 (14.2)	
Histology					.51
Squamous cell carcinoma	519 (16.5)	358 (16.2)	71 (15.3)	90 (19.4)	
Adenocarcinoma	2490 (79.2)	1765 (79.7)	369 (79.5)	356 (76.6)	
Large-cell carcinoma	16 (0.5)	10 (0.5)	3 (0.6)	3 (0.6)	
NSCLC NOS^a^	234 (7.4)	164 (7.4)	40 (8.6)	30 (6.5)	
Stage^b^					.83
1	1113 (35.4)	802 (36.2)	161 (34.7)	168 (36.1)	
2	276 (8.8)	190 (8.6)	40 (8.6)	46 (9.9)	
3	455 (14.5)	328 (14.8)	61 (13.1)	66 (14.2)	
4	1282 (40.8)	895 (40.4)	202 (43.5)	185 (39.8)	
Gene mutation					
EGFR^c^	1131 (36.0)	799 (36.1)	166 (35.8)	166 (35.7)	.98
ALK^d^	209 (6.6)	159 (7.2)	27 (5.8)	23 (4.9)	.16

a NSCLC NOS: non–small-cell lung cancer not otherwise specified.

b Stage was classified according to the eighth edition of the American Joint Committee on Cancer staging system.

c EGFR: epidermal growth factor receptor.

d ALK: anaplastic lymphoma kinase.

Absolute SMD values for baseline characteristics between included and excluded patients are shown in Table S2 in Multimedia Appendix 1. Excluded patients had higher 5-year mortality than included patients (3793/5520, 68.7% vs 1794/3144, 57.1%; SMD 0.241) and were older (mean 68.4, SD 10.7 vs 65.5, SD 10.7 years; SMD 0.278).

### Training Models and Performance Evaluation

We selected 5 distinct hyperparameter configurations from the Hyperband optimization (Table S3 in Multimedia Appendix 1). For each configuration, we rebuilt the network using the corresponding tuned settings, retrained it on the training cohort, and evaluated its performance on both the training and test sets. During this process, we recorded a comprehensive set of performance metrics to facilitate direct comparisons across models. Each final model was serialized into a Keras file to ensure reproducibility and enable downstream inference. Each file contained the complete network architecture, learned weights, and optimizer state, allowing the model to be reloaded and applied with a single command in any compatible Python environment.

**Figure 4.**
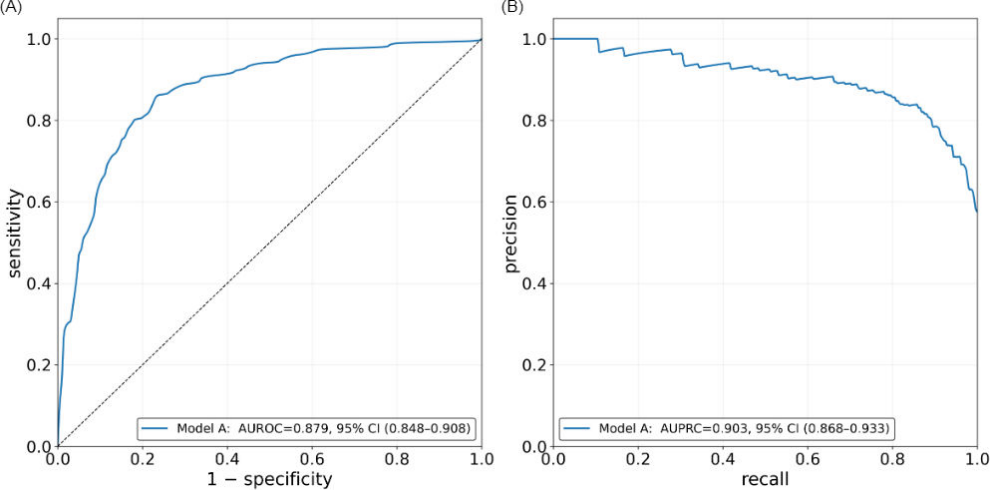
Discrimination performance of model A on the test set: (A) receiver operating characteristic curve and (B) precision-recall curve. AUPRC: area under the precision-recall curve; AUROC: area under the receiver operating characteristic curve.

**Figure 5.**
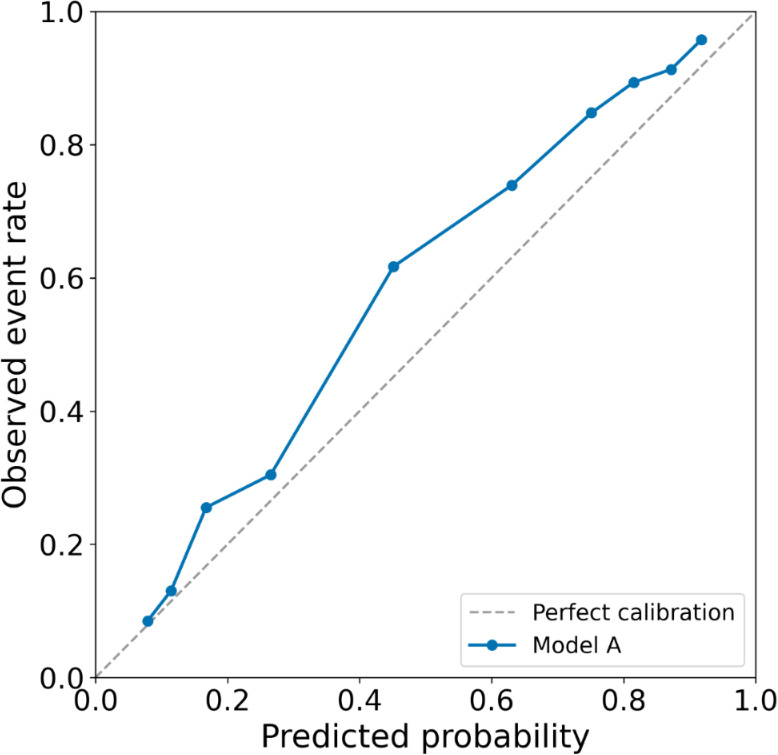
Calibration plot of model A on the test set. The dashed diagonal line indicates perfect calibration.

Of the 5 models, we designated model A as the primary model for reporting. On the held-out test set, model A achieved an AUC of 0.879 (95% CI 0.848‐0.908), with an accuracy of 0.806 (95% CI 0.774‐0.841) and an *F*_1_-score of 0.824 (95% CI 0.788‐0.858). The AUC and AUPRC of model A are shown in Figure 4. Model A also demonstrated reasonable calibration on the test set (Figure 5), with a Brier score of 0.142. Model U (unknown-aware encoding) achieved a test AUC of 0.865 (95% CI 0.830‐0.897), corresponding to a small decrease compared with model A (ΔAUC=−0.014, 95% CI −0.025 to −0.003). Other discrimination and classification metrics showed similarly small differences (Table S4 in Multimedia Appendix 1).

Figures S1 and S2 in Multimedia Appendix 1 show the discrimination performance and calibration curves of the 5 models on the test set. Across models A to E, performance was highly consistent, with AUC ranging from 0.875 to 0.879 and AUPRC ranging from 0.894 to 0.903. The CPH model exhibited similar performance, with an AUC of 0.878 (95% CI 0.844‐0.908). The TNM-only CPH benchmark model yielded a lower test AUC of 0.845 (95% CI 0.806‐0.880). Table S5 in Multimedia Appendix 1 provides the complete set of discrimination and classification metrics for all models, including both CPH baselines (full and TNM only). Brier scores for models A to E were also similar (approximately 0.140; Table S6 in Multimedia Appendix 1), indicating comparable calibration across model variants.

We evaluated within-stage discrimination for the primary model (model A). Within-stage performance was generally clinically meaningful in stages 1, 3, and 4 (AUC of 0.753, 95% CI 0.653‐0.844 in stage 1; 0.722, 95% CI 0.569‐0.855 in stage 3; and 0.725, 95% CI 0.625‐0.825 in stage 4). Stage 2 exhibited the lowest AUC and a wide 95% CI (0.642, 95% CI 0.471‐0.796). Detailed within-stage performance metrics for models A to E are summarized in Table S7 in Multimedia Appendix 1.

### Feature Permutation and Changes in AUC

**Figure 6.**
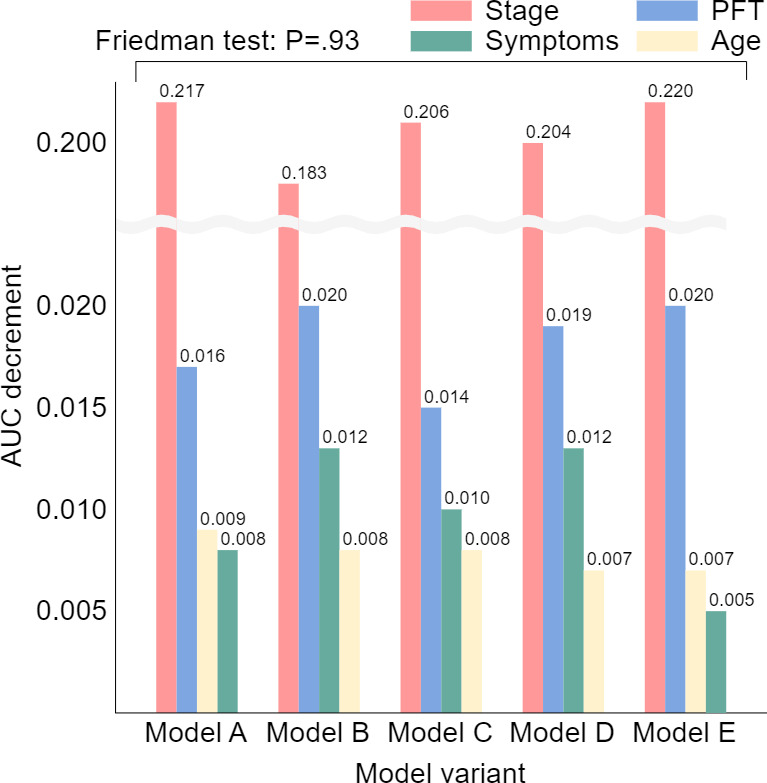
Mean decrease in test set area under the receiver operating characteristic curve (AUC) after grouped feature permutation across the 5 model variants (models A-E). Stage produced the largest decrease in AUC, followed by pulmonary function test (PFT), symptoms, and age, with consistent rankings across model variants (Friedman test: *P*=.93).

We conducted grouped-feature permutation analyses to assess feature importance. Stage yielded the largest decrease in AUC (0.217, 95% CI 0.212‐0.221), indicating this feature’s predominant contribution. PFT ranked second (0.016, 95% CI 0.015‐0.017), followed by age (0.009, 95% CI 0.009‐0.010) and symptoms (0.008, 95% CI 0.007‐0.008). The permutation-based importance hierarchy was highly consistent across the 5 model variants (Figure 6 and Table S8 in Multimedia Appendix 1). A Friedman test showed no statistically significant differences in importance rankings across models (*P*=.93).

Feature importance was analyzed separately by histologic subtype (Table S9 in Multimedia Appendix 1). Discrimination was higher in the adenocarcinoma cohort than in the squamous cell carcinoma cohort. Within adenocarcinoma, gene mutation showed a larger decrease in AUC than in the overall analysis (0.003, 95% CI 0.003‐0.003), a pattern that was consistent across all 5 model variants. This pattern was not observed in the squamous cell carcinoma cohort.

## Discussion

### Overall Findings

Using the nationwide KCCR lung cancer cohort, we developed and validated a grouped-input deep learning model to predict 5-year mortality in NSCLC. We ensured reproducibility and minimized data leakage by implementing stratified training, validation, and test splits, with all preprocessing confined to the training set. On the test set, model A demonstrated strong discrimination and reasonable calibration. Across the 5 Hyperband-tuned model variants, test set performance (AUC=0.875‐0.879) and grouped-feature permutation profiles were highly consistent, supporting the robustness of the proposed framework.

### Model Performance

The grouped-input deep learning model demonstrated statistically comparable discrimination to that of a conventional CPH model built on the same routine clinical variables. This finding suggests that, for low-dimensional tabular clinical data without multimodal integration, complex neural network architectures may offer limited incremental benefit over interpretable regression-based approaches [17,18]. Taken together, these results reinforce that model complexity should be guided by data structure rather than architectural novelty. In this context, our findings are best interpreted as a methodological comparison rather than evidence that deep learning is inherently superior for 5-year mortality prediction using registry-based clinical features alone. Nevertheless, deep learning architectures can model nonlinear interactions and readily integrate radiological or molecular data. Consistency across the 5 Hyperband-tuned model variants supports the robustness of the preprocessing pipeline, suggesting that the proposed framework provides a stable foundation for future multimodal extensions.

Importantly, our model is a baseline prognostic model and does not explicitly incorporate treatment exposures, which can substantially modify survival. Thus, predicted risk represents an average prognosis across heterogeneous treatment pathways in the cohort and should not be interpreted as a treatment-conditional estimate.

Although the TNM-only benchmark yielded lower overall discrimination (AUC=0.845, 95% CI 0.806‐0.880), we further assessed model performance within each stage to evaluate whether nonstaging variables retained discriminatory value when staging information was held constant. Our within-stage analysis indicates that the model provides prognostic discrimination beyond TNM staging alone. Within-stage performance was favorable in stages 1, 3, and 4 (AUCs generally of >0.700). In contrast, discrimination in stage 2 was lower and imprecise, with wide CIs that in some cases crossed 0.50, likely reflecting the small sample size in this subgroup. This may also partly reflect unmeasured heterogeneity in treatment pathways within stage 2, which could not be explicitly modeled due to incomplete treatment data. Prior validations of the TNM system have shown that revisions from the seventh to the eighth and ninth editions of the American Joint Committee on Cancer staging system yielded only modest gains in discriminatory ability and overall prognostic separation remained limited [19,20]. Although the model demonstrated incremental prognostic value beyond stage in the overall cohort, discrimination within stage 2 was modest (AUC=0.642). The relatively small sample size of stage 2 patients may have contributed to wider CIs and statistical instability; however, even accounting for this uncertainty, the current performance suggests that the model is not sufficiently robust for clinical risk stratification in stage 2 NSCLC. Additional prognostic variables and external validation would likely be required to improve reliability in this subgroup.

Recent studies have used diverse machine learning techniques to predict lung cancer survival, with reported AUCs ranging from approximately 0.650 to 0.800 [11,21]. In many of these investigations, tree-based ensembles such as random forests and Extreme Gradient Boosting reported stronger discrimination than simple deep learning methods [22-24]. Nevertheless, because of their ability to integrate heterogeneous inputs, researchers continue to explore deep learning models in NSCLC. For example, Lai et al [25] combined biomarker and clinical data, achieving an AUC of 0.816 with an accuracy of 0.754. Wang et al [26] reported an AUC of 0.715 using a similar multimodal design. In contrast, Hindocha et al [23] developed an ensemble model based solely on clinical variables, which reached an AUC of 0.717. By comparison, our models—despite relying exclusively on routine clinical features—produced AUCs exceeding 0.875 and accuracies of approximately 0.800. These differences may reflect a combination of factors, including the grouped-input design (which mitigates sparsity, shares weights within clinically coherent groups, and captures nonlinear relationships) [27,28], rigorous leakage control, and large multicenter settings. Deep learning models trained on substantially smaller cohorts frequently show weaker discrimination and greater overfitting tendencies [11]. However, cross-study comparisons should be interpreted cautiously due to heterogeneity in cohorts, end points, and validation protocols.

### Permutation-Based Feature Importance

Permutation-based importance analysis showed that stage was the dominant predictor of mortality, followed by PFT, symptoms, and age, consistent with earlier studies [29-33]. Because permutation importance can undervalue variables that are correlated with a single dominant predictor, this dominance may have led to a relative underestimation of other clinically relevant features [21,34,35]. To minimize such bias, variables were permuted at the group level rather than individually [36]. However, interpretation requires caution. For example, PFT included not only spirometric measurements (forced vital capacity, forced expiratory volume in 1 second, and diffusion capacity of the lungs for carbon monoxide) but also indicators of test availability. Given our sentinel-based encoding for unmeasured PFTs, the apparent importance of PFT may partly capture care process information (eg, workup completeness or surgical candidacy) rather than the prognostic effect of lung function values themselves. This likely reflects a characteristic of real-world registry data, in which diagnostic testing patterns may be correlated with clinical severity and treatment eligibility. Accordingly, feature importance in this context should not be interpreted as reflecting the independent prognostic effect of spirometry alone but rather as a combination of lung function measurements and care-related factors.

Smoking-related variables and histology exhibited only modest marginal importance, which is consistent with the feature distribution imbalance of the cohort (a high prevalence of never smokers and adenocarcinoma), reducing variability in smoking metrics and limiting representation of squamous or large-cell carcinoma [37,38]. The relatively low importance of BMI is compatible with the reported “obesity paradox,” in which risk appears nonlinear and a single baseline measurement may dilute the marginal effect [39,40]. We also evaluated whether model-level factors could bias these rankings. The 5 models were trained using distinct hyperparameter settings, including different dropout rates and different numbers of hidden units in the feature group–specific layers. Although such differences can influence importance estimates [41], the rankings were highly concordant across models, suggesting that any bias from hyperparameter choices was negligible.

This study examined the importance of gene mutations within histological subgroups. In adenocarcinoma, gene mutation became modestly more influential than in the overall analysis, with EGFR consistently surpassing ALK across all 5 models. In contrast, neither mutation provided meaningful predictive value in squamous cell carcinoma. Although both EGFR and ALK have clear therapeutic relevance in NSCLC as targets of tyrosine kinase inhibitors, their apparent prognostic contributions can vary by histology and prevalence [2,42]. Our findings align with those of previous reports in which EGFR was associated with more favorable outcomes in adenocarcinoma, whereas ALK mutations were less frequent and exhibited weaker associations with survival [42-44]. Because squamous cell carcinoma rarely harbors either mutation and is associated with intrinsically poorer outcomes [44], effect estimates in this subgroup are inherently uncertain. Moreover, residual confounding by therapy exposure cannot be excluded in the absence of treatment-level data. Different patterns may emerge in cohorts with larger proportions of squamous cell carcinoma or higher ALK mutation frequencies.

### Limitations

This study has several limitations. First, it was a retrospective analysis limited to a Korean cohort; interethnic differences in EGFR mutation prevalence, histology, and smoking patterns may affect generalizability [45,46]. Second, registry-based data have inherent constraints. Because our cohort was diagnosed between 2014 and 2017, key biomarkers (eg, KRAS mutation, programmed death-ligand 1, and tumor mutational burden) and detailed treatment variables were not routinely available or consistently recorded. As a result, the absolute mortality estimates generated by our model may be higher than those observed under contemporary treatment standards, and model calibration may shift in more recent patient populations. Therefore, external validation and potential recalibration in temporally distinct cohorts will be necessary before clinical implementation. Third, “unknown” values for ECOG status and smoking history were recoded to default categories to enable complete input vectors. If these values were not missing at random, this preprocessing step might have introduced optimistic bias in survival prediction. Fourth, although the overall sample size was large, some subgroup analyses were underpowered. Notably, the stage 2 subgroup in the test set was small (46/465, 9.9%) with wide CIs, some of which crossed 0.50. This is particularly important because stage 2 represents a key clinical decision point for adjuvant therapy. Fifth, although we compared our model with a conventional CPH model, we did not conduct extensive benchmarking against multiple strong tabular learners; therefore, comparative performance across alternative tabular modeling approaches should be interpreted cautiously. Because TNM stage dominated permutation-based importance, residual correlation-related bias cannot be excluded and may attenuate the apparent importance of correlated predictors. Finally, our evaluation relied on internal validation within a single registry split. As a result, the transportability of the model across different health care systems, patient populations, and treatment eras remains uncertain. Given these constraints, external validation in an independent cohort is required to assess both discrimination and calibration stability before clinical deployment or widespread adoption.

### Conclusions

In this study, a deep learning model based on the nationwide lung cancer staging dataset from the KCCR demonstrated performance comparable to that of a conventional CPH model and previously reported machine learning methods. By aligning inputs with clinically coherent groups and quantifying contributions through permutation-based, group-level importance, the framework provides transparent and reproducible explanations using routine clinical variables. These features position the model as a practical foundation for future clinical evaluation and integration of additional modalities such as imaging or molecular data in more complex predictive settings. Future work should include external validation in independent and temporally distinct cohorts as well as prospective evaluation of clinical utility before routine clinical implementation.

## Supplementary material

10.2196/80574 Multimedia Appendix 1 Consolidated supplementary tables for this study, including cohort description, preprocessing and variable definitions, model configuration, and full evaluation outputs. These materials support the main results and are provided for transparency and reproducibility.

## Abbreviations

ALK: anaplastic lymphoma kinase
AUC: area under the receiver operating characteristic curve
AUPRC: area under the precision-recall curve
CPH: Cox proportional hazards
ECOG: Eastern Cooperative Oncology Group
EGFR: epidermal growth factor receptor
KCCR: Korea Central Cancer Registry
MLP: multilayer perceptron
NSCLC: non–small-cell lung cancer
PFT: pulmonary function test
SMD: standardized mean difference
TNM: tumor, node, metastasis
