# Supplementary material for: Predicting 5-Year Mortality in Non–Small-Cell Lung Cancer Using the Korean Central Cancer Registry: Model Development and Validation Study
Source: JMIR Med Inform. 2026 Jun 8;14:e80574. doi: 10.2196/80574 (PMC13245844; doi:10.2196/80574)
Supplement: Multimedia Appendix 1 [file medinform-v14-e80574-s001.docx]

# **Supplementary Tables and Figures for:**

# **Predicting 5-Year Mortality in NSCLC: Development and Validation of a Deep Learning Model Using the Korean Central Cancer Registry**

Jong Hyuk Lee^1^, Ho Cheol Kim^2^, Kyu-Won Jung^3^, Chang Min Choi^1,2^

^1^Department of Oncology, Asan Medical Center, University of Ulsan College of Medicine, Seoul, Republic of Korea

^2^Department of Pulmonary and Critical Care Medicine, Asan Medical Center, University of Ulsan College of Medicine, Seoul, Republic of Korea

^3^Division of Cancer Registration and Surveillance, National Cancer Control Institute, National Cancer Center, Goyang, Korea

**Corresponding Author:**

Chang Min Choi, MD, PhD

Department of Pulmonary and Critical Care Medicine

Asan Medical Center, University of Ulsan College of Medicine

88, Olympic-ro 43-gil, Songpa-gu, Seoul, 05505, Republic of Korea

Phone: +82-10-9792-9607, Email: ccm@amc.seoul.kr

**Supplementary Table 1**. Mapping of original registry variables to preprocessed model inputs and coding rules.

|  | **Original variables** | **Preprocessed variables** |
| --- | --- | --- |
|  |  |  |
| **Age** | Patient’s age at the time of diagnosis was expressed in whole years (integers). | Used without modification. |
| **Gender** | 1: male, 2: female. | Used without modification. |
| **BMI^a^** | height: 100–250 cm, weight: 30–200 kg. | Calculated from original data. |
| **ECOG^b^** | Values range 0–5, with 9 indicating ‘unknown’. | 9: unknown was treated as ECOG score 0. |
| **Symptoms** | 1: asymptomatic, 2: cough, 3: sputum, 4: dyspnea, 5: hoarseness, 6: hemoptysis, 7: weight loss, 8: pain, 9: others. | New columns, labeled ‘symptom_2’ through ‘symptom_9’, were created. Each column is coded as 0: no symptom and 1: presence of that symptom. If a patient was asymptomatic, then all ‘symptom_2’ to ‘symptom_9’ values were 0. |
| **Smoke** | ‘Smoke’ indicates smoke history.  1: never smoker, 2: current smoker, 3: ex-smoker (quit >1 year ago), 9: unknown. | 9: unknown was treated as never smoker. |
|  | ‘smokedose’: packs smoked per day. | If ‘smoke’ was 1, ‘smokedose’ was set to 0. |
|  | ‘smokedur’: total years of smoking. | If ‘smoke’ was 1, ‘smokedur’ was set to 0. |
|  | ‘smokequityr’: the year the patient quit smoking. | ‘smokequitdur’: newly derived variable representing the duration of smoking cessation.  If ‘smoke’ was 1, ‘smokequitdur’ was set to −1.  If ‘smoke’ was 2, ‘smokequitdur’ was set to 0.  If ‘smoke’ was 3, ‘smokequitdur’ was calculated as the difference between ‘smokequityr’ and either 2023 (the last follow-up year) or the year of death. |
| **PFT^c^** | ‘pft’: indicates whether PFT was performed.  0: not performed, 1: performed. | Used without modification. |
|  | ‘fvc^d^’: FVC measured in liters.  ‘fev1^e^’: FEV_1_ measured in litters.  ‘DLCO^f^’: value of DLCO. | If ‘pft’ was 0, ‘fvc’, ‘fev1’, and ‘DLCO’were set to −1. |
| **Histology** | ‘h_name_1’: squamous cell carcinoma, ‘h_name_2’: adenocarcinoma, ‘h_name_3’: large cell carcinoma, ‘h_name_4’: NSCLC NOS^g^.  0: negative, 1: positive. | Used without modification. |
| **Stage^h^** | ‘hc_t’ represents clinical T stage.  0: Tx, 1: T1a, 2: T1b, 3: T1 NOS, 4: T2a, 5: T2b, 6: T2 NOS, 7: T3, 8: T4. | Used without modification. |
|  | ‘hc_n’ represents clinical N stage.  0: N0, 1: N1, 2: N2, 3: N3. | Used without modification. |
|  | ‘hc_m’ represents clinical M stage.  0: M0, 1: M1a, 2: M1b, 3: M1 NOS. | Used without modification. |
| **Gene mutation** | ‘egfr_mutation’ and ‘alk_ihc’ each indicate whether the patient has an *EGFR^i^* or *ALK^j^* mutation, respectively.  0: negative, 1: positive, 9: not performed. | Patients missing results for either gene mutation were excluded. |
| **5-year mortality and survival** | ‘h_date’: diagnosed date by biopsy. (YYYY-MM)  ‘death_date’: date of death, blank if patient was not dead. (YYYY-MM-DD) | ‘death_date’ was divided into 3 groups.  Day 1–10: same calendar month as recorded  Day 11–20: 0.5 month was added  Day 21–31: rounded up to the first day of the following month  After adjusting ‘death_date’ as above, we calculated the interval between the adjusted ‘death_date’ and ‘h_date’.  Five-year survivors were defined as patients confirmed to be alive at ≥60 months after the diagnosis date based on nationwide mortality ascertainment. Patients who died within 60 months were classified as non-survivors. 'surv_5yr' = 1 indicates death within 5 years (event = 1), and 'surv_5yr' = 0 indicates survival beyond 5 years. |

^a^BMI, body mass index

^b^ECOG, Eastern Cooperative Oncology Group performance status

^c^PFT, pulmonary function test

^d^FVC, forced vital capacity

^e^FEV_1_, forced expiratory volume in one second

^f^DLCO, diffusion capacity of the lung for carbon monoxide

^g^NSCLC NOS, non-small cell lung cancer not otherwise specified

^h^Stage was classified according to the 8th edition of the AJCC staging system.

^i^EGFR, epidermal growth factor receptor

^j^ALK, anaplastic lymphoma kinase.

**Supplementary Table 2.** Baseline characteristics of included vs. excluded patients and |SMD|^a^.

|  | **Included patients (N = 3,144)** | **Excluded patients (N = 5,520)** | **\|SMD\|** |
| --- | --- | --- | --- |
| **Sex, n (%)** |  |  | 0.271 |
| Male | 1908 (60.7%) | 4054 (73.4%) |  |
| Female | 1236 (39.3%) | 1466 (26.6%) |  |
| **Age (years), mean (SD)** | 65.5 (10.7) | 68.4 (10.7) | 0.278 |
| **BMI^b^ (kg/m^2^), mean (SD)** | 23.4 (3.3) | 22.9 (3.4) | 0.148 |
| **ECOG^c^, n (%)** |  |  | 0.106 |
| 0 | 1902 (60.5%) | 3182 (57.6%) |  |
| 1 | 1023 (32.5%) | 1767 (32.0%) |  |
| 2 | 160 (5.1%) | 343 (6.2%) |  |
| 3 | 45 (1.4%) | 165 (3.0%) |  |
| 4 | 14 (0.4%) | 54 (1.0%) |  |
| **Smoke, n (%)** |  |  | 0.204 |
| Never smoked | 1420 (45.2%) | 1912 (34.6%) |  |
| Current smoker | 927 (29.5%) | 1909 (34.6%) |  |
| Ex-smoker | 797 (25.3%) | 1699 (30.8%) |  |
| **PFT^d^, mean (SD)** |  |  |  |
| FVC^e^ (L) | 3.2 (1.5) | 3.1 (2.0) | 0.042 |
| FEV_1_^f^ (L) | 2.3 (1.3) | 2.5 (16.8) | 0.014 |
| DLCO^g^ (%) | 85.2 (22.1) | 79.7 (23.9) | 0.238 |
| **5-year mortality^h^** | 1794 (57.1%) | 3793 (68.7%) | 0.241 |

^a^ |SMD|, absolute standardized mean differences

^b^BMI, body mass index

^c^ECOG, Eastern Cooperative Oncology Group performance status

^d^PFT, pulmonary function test

^e^FVC, forced vital capacity

^f^FEV_1_, forced expiratory volume in one second

^g^DLCO, diffusion capacity of the lung for carbon monoxide

^h^For excluded patients who lacked an exact diagnosis date, 5-year mortality was estimated using the registered diagnosis year by assuming January 1 of that year as the diagnosis date.

**Supplementary Table 3**. Hyperparameter configurations selected by Hyperband optimization for the five model variants (Models A–E).^a^

|  | **Model A** | **Model B** | **Model C** | **Model D** | **Model E** |
| --- | --- | --- | --- | --- | --- |
|  |  |  |  |  |  |
| **Dropout** |  |  |  |  |  |
| Smoke | 0.40 | 0.40 | 0.35 | 0.40 | 0.35 |
| PFT | 0.45 | 0.50 | 0.45 | 0.45 | 0.40 |
| Histology | 0.35 | 0.40 | 0.40 | 0.40 | 0.40 |
| Stage | 0.30 | 0.30 | 0.45 | 0.50 | 0.45 |
| Gene mutation | 0.45 | 0.45 | 0.40 | 0.35 | 0.30 |
| Main layer | 0.35 | 0.50 | 0.50 | 0.40 | 0.30 |
| **Units** |  |  |  |  |  |
| Smoke | 16 | 4 | 8 | 4 | 8 |
| PFT^b^ | 8 | 8 | 8 | 8 | 16 |
| Histology | 4 | 16 | 8 | 4 | 4 |
| Stage | 8 | 4 | 8 | 16 | 16 |
| Gene mutation | 16 | 8 | 16 | 16 | 32 |
| Main layer | 128 | 128 | 128 | 128 | 64 |
| **Training** |  |  |  |  |  |
| Learning rate | 6.84×10^-3^ | 9.36×10^-3^ | 4.99×10^-3^ | 8.31×10^-3^ | 7.47×10^-3^ |
| λ for L2 | 5.00×10^-3^ | 3.90×10^-3^ | 2.78×10^-4^ | 2.17×10^-4^ | 2.70×10^-4^ |

^a^Optimal dropout rates, number of units, learning rate, and L2 regularization constants for each of the five final models.

^b^PFT, pulmonary function test.

**Supplementary Table 4.** Test set performance of Model A and Model U^a^ (unknown-aware encoding sensitivity analysis).^b^

|  | **Model A** | **Model U** | **Δ (U − A)^c^** |
| --- | --- | --- | --- |
| AUC^d^ | 0.879 (0.848–0.908) | 0.865 (0.830–0.897) | -0.014 (-0.025–-0.003) |
| Accuracy | 0.806 (0.774–0.841) | 0.800 (0.763–0.834) | -0.006 (-0.030–0.019) |
| F1 score | 0.824 (0.788–0.858) | 0.824 (0.785–0.858) | -0.001 (-0.023–0.022) |
| Precision | 0.861 (0.821–0.902) | 0.835 (0.786–0.878) | -0.027 (-0.054–-0.002) |
| Recall | 0.790 (0.741–0.837) | 0.813 (0.766–0.857) | 0.022 (-0.008–0.054) |

^a^Model U is a sensitivity model in which ‘Unknown’ ECOG performance status and smoking history were retained as distinct categories (i.e., not merged into ECOG 0 or never smoker). Model U was trained using the same architecture and hyperparameter settings as Model A.

^b^95% CIs were derived from 1,000 bootstrap resamples.

^c^Δ indicates the difference between Model U and Model A (U − A).

^d^AUC, area under the receiver operating characteristic curve

**Supplementary Table 5**. Discrimination and classification performance metrics of Models A–E on the training, validation, and test sets.^a^

|  | **Model A** | **Model B** | **Model C** | **Model D** | **Model E** | **CPH^b^** | **CPH, Stage-only** |
| --- | --- | --- | --- | --- | --- | --- | --- |
| **Training set (N = 2,215)** | | | | | | |  |
| AUC^c^ | 0.890 (0.877–0.904) | 0.887 (0.873–0.902) | 0.892 (0.878–0.905) | 0.891 (0.878–0.905) | 0.892 (0.879–0.906) | 0.883 (0.869–0.897) | 0.851 (0.836–0.867) |
| Accuracy | 0.816 (0.800–0.832) | 0.818 (0.803–0.834) | 0.816 (0.800–0.832) | 0.821 (0.806–0.837) | 0.816 (0.800–0.833) | 0.813 (0.796–0.829) | 0.784 (0.767–0.800) |
| F1 score | 0.829 (0.813–0.845) | 0.836 (0.821–0.852) | 0.838 (0.822–0.853) | 0.840 (0.824–0.855) | 0.831 (0.815–0.848) | 0.833 (0.817–0.849) | 0.800 (0.784–0.817) |
| Precision | 0.877 (0.858–0.896) | 0.857 (0.836–0.877) | 0.839 (0.818–0.858) | 0.858 (0.838–0.877) | 0.871 (0.851–0.891) | 0.846 (0.827–0.866) | 0.842 (0.822–0.863) |
| Recall | 0.786 (0.764–0.808) | 0.816 (0.796–0.837) | 0.838 (0.817–0.857) | 0.823 (0.803–0.844) | 0.795 (0.773–0.817) | 0.820 (0.800–0.840) | 0.763 (0.740–0.785) |
| **Validation set (N = 464)** | | | | | | |  |
| AUC | 0.910 (0.882–0.938) | 0.909 (0.880–0.936) | 0.906 (0.878–0.934) | 0.908 (0.879–0.936) | 0.906 (0.878–0.934) | 0.912 (0.844–0.935) | 0.881 (0.848–0.911) |
| Accuracy | 0.828 (0.793–0.862) | 0.834 (0.800–0.866) | 0.843 (0.808–0.877) | 0.841 (0.806–0.873) | 0.836 (0.802–0.871) | 0.834 (0.797–0.866) | 0.806 (0.767–0.843) |
| F1 score | 0.844 (0.809–0.877) | 0.853 (0.823–0.884) | 0.863 (0.831–0.896) | 0.860 (0.828–0.892) | 0.852 (0.819–0.884) | 0.853 (0.818–0.885) | 0.824 (0.788–0.859) |
| Precision | 0.879 (0.837–0.918) | 0.868 (0.826–0.908) | 0.865 (0.822–0.905) | 0.867 (0.825–0.908) | 0.890 (0.849–0.927) | 0.868 (0.824–0.905) | 0.861 (0.815–0.904) |
| Recall | 0.813 (0.764–0.855) | 0.839 (0.793–0.881) | 0.862 (0.819–0.902) | 0.854 (0.808–0.895) | 0.817 (0.771–0.861) | 0.839 (0.791–0.882) | 0.790 (0.741–0.839) |
| **Test set (N = 465)** | | | | | | |  |
| AUC | 0.879 (0.848–0.908) | 0.876 (0.844–0.906) | 0.875 (0.843–0.905) | 0.878 (0.846–0.908) | 0.877 (0.845–0.906) | 0.878 (0.844–0.908) | 0.845 (0.806–0.880) |
| Accuracy | 0.806 (0.774–0.841) | 0.813 (0.781–0.847) | 0.822 (0.789–0.856) | 0.819 (0.789–0.854) | 0.796 (0.761–0.830) | 0.826 (0.791–0.860) | 0.776 (0.740–0.813) |
| F1 score | 0.824 (0.788–0.858) | 0.834 (0.803–0.868) | 0.846 (0.816–0.876) | 0.842 (0.811–0.874) | 0.815 (0.781–0.848) | 0.849 (0.813–0.882) | 0.793 (0.753–0.829 |
| Precision | 0.861 (0.821–0.902) | 0.846 (0.804–0.887) | 0.838 (0.795–0.880) | 0.842 (0.802–0.884) | 0.846 (0.803–0.887) | 0.847 (0.801–0.888) | 0.847 (0.800–0.891) |
| Recall | 0.790 (0.741–0.837) | 0.824 (0.780–0.867) | 0.855 (0.812–0.894) | 0.843 (0.799–0.885) | 0.787 (0.740–0.833) | 0.850 (0.806–0.895) | 0.745 (0.694–0.795) |

^a^95% CI were derived from 1,000 bootstrap resamples.

^b^CPH, Cox proportional hazard model.

^c^AUC, area under the receiver operating characteristic curve

**Supplementary Table 6.** Brier scores of Models A–E on the test set.

|  | Model A | Model B | Model C | Model D | Model E | CPH |
| --- | --- | --- | --- | --- | --- | --- |
| Brier score | 0.142 | 0.143 | 0.138 | 0.139 | 0.143 | 0.141 |

**Supplementary Table 7**. Within-stage discrimination performance of Models A–E on the test set.^a^

|  | **Model A** | **Model B** | **Model C** | **Model D** | **Model E** | **CPH^b^** |
| --- | --- | --- | --- | --- | --- | --- |
| Baseline AUC^c^ | 0.879 (0.848–0.908) | 0.876 (0.844–0.906) | 0.875 (0.843–0.905) | 0.878 (0.846–0.908) | 0.877 (0.845–0.906) | 0.878 (0.844–0.908) |
| Stage Ⅰ (N = 168) | 0.753 (0.653–0.844) | 0.737 (0.623–0.836) | 0.739 (0.632–0.840) | 0.751 (0.645–0.848) | 0.733 (0.626–0.834) | 0.768 (0.663–0.856) |
| Stage Ⅱ (N = 46) | 0.642 (0.471–0.796) | 0.686 (0.523–0.826) | 0.635 (0.459–0.794) | 0.645 (0.469–0.805) | 0.644 (0.475–0.797) | 0.634 (0.460–0.788) |
| Stage Ⅲ (N = 66) | 0.722 (0.569–0.855) | 0.746 (0.602–0.872) | 0.729 (0.586–0.859) | 0.717 (0.572–0.846) | 0.724 (0.576–0.856) | 0.759 (0.610–0.883) |
| Stage Ⅳ (N = 185) | 0.725 (0.625–0.825) | 0.701 (0.603–0.798) | 0.702 (0.592–0.810) | 0.717 (0.610–0.821) | 0.729 (0.633–0.827) | 0.747 (0.622–0.852) |

^a^95% CI were derived from 1,000 bootstrap resamples.

^b^CPH, Cox proportional hazard model.

^c^AUC, area under the receiver operating characteristic curve

**Supplementary Table 8**. Permutation-based feature-group importance rankings based on the AUC^a^ decrease across Models A–E.^b^

| **Rank** | **Model A** | **Model B** | **Model C** | **Model D** | **Model E** |
| --- | --- | --- | --- | --- | --- |
| 1 | Stage: 0.217  (0.212–0.221) | Stage: 0.183  (0.178–0.187) | Stage: 0.206  (0.202–0.211) | Stage: 0.204  (0.200–0.209) | Stage: 0.220  (0.215–0.224) |
| 2 | PFT^c^: 0.016  (0.015–0.017) | PFT: 0.020  (0.019–0.021) | PFT: 0.014  (0.013–0.015) | PFT: 0.019  (0.018–0.020) | PFT: 0.020  (0.019–0.021) |
| 3 | Age: 0.009  (0.009–0.010) | Symptoms: 0.012  (0.012–0.013) | Symptoms: 0.010  (0.009–0.011) | Symptoms: 0.012  (0.012–0.013) | Age: 0.007  (0.007–0.008) |
| 4 | Symptoms: 0.008  (0.007–0.008) | Age: 0.008  (0.007–0.008) | Age: 0.008  (0.007–0.009) | Age: 0.007  (0.007–0.008) | Symptoms: 0.005  (0.004–0.005) |
| 5 | ECOG^d^: 0.004  (0.003–0.004) | ECOG: 0.007  (0.006–0.007) | ECOG: 0.005  (0.005–0.006) | ECOG: 0.005  (0.005–0.006) | ECOG: 0.003  (0.003–0.004) |
| 6 | Gender: 0.003  (0.002–0.003) | Gender: 0.003  (0.002–0.003) | Gender: 0.002  (0.002–0.003) | Gene mutation: 0.002  (0.002–0.003) | Gender: 0.003  (0.003–0.004) |
| 7 | Gene mutation: 0.002  (0.002–0.002) | Gene mutation: 0.001  (0.001–0.002) | Gene mutation: 0.002  (0.002–0.002) | Gender: 0.002  (0.002–0.002) | Gene mutation: 0.002  (0.002–0.003) |
| 8 | Histology: 0.001  (0.000–0.001) | Smoke: 0.000  (0.000–0.000) | Smoke: 0.001  (0.001–0.001) | Histology: 0.001  (0.001–0.001) | Smoke: 0.001  (0.001–0.001) |
| 9 | Smoke: 0.000  (0.000–0.000) | Histology: 0.000  (-0.000–0.000) | BMI: 0.000  (-0.000–0.000) | Smoke: 0.000  (0.000–0.001) | Histology: 0.000  (-0.000–0.000) |
| 10 | BMI^e^: 0.000  (0.000–0.000) | BMI: -0.000  (-0.000–-0.000) | Histology: 0.000  (-0.001–-0.000) | BMI: 0.000  (0.000–0.000) | BMI: 0.000  (-0.000–-0.000) |

^a^AUC, area under the receiver operating characteristic curve.

^b^For each feature group, we performed 100 permutations to obtain the AUC decrement distribution (baseline AUC − permuted AUC) and estimated the 95 % CI by bootstrapping these drops 1,000 times. Friedman test showed no meaningful difference in feature importance ranking (*p* = .928).

^c^PFT, pulmonary function test

^d^ECOG, Eastern Cooperative Oncology Group performance status

^e^BMI, body mass index

**Supplementary Table 9**. Permutation-based importance of 'Gene mutation (*EGFR^a^* / *ALK^b^*)' group in adenocarcinoma and squamous cell carcinoma subgroups across Models A–E.^c^

|  | **Model A** | **Model B** | **Model C** | **Model D** | **Model E** |
| --- | --- | --- | --- | --- | --- |
|  |  |  |  |  |  |
| **Adenocarcinoma (N = 356)** | | | | | |
| Baseline AUC^d^ | 0.889 (0.853–0.922) | 0.886 (0.850–0.919) | 0.886 (0.850–0.919) | 0.889 (0.852–0.922) | 0.887 (0.851–0.920) |
| **AUC decrement** | | | | | |
| Gene mutation | 0.003 (0.003–0.003) | 0.002 (0.002–0.003) | 0.004 (0.003–0.004) | 0.004 (0.003–0.004) | 0.003 (0.003–0.004) |
| EGFR | 0.002 (0.002–0.003) | 0.002 (0.002–0.002) | 0.002 (0.002–0.003) | 0.002 (0.002–0.002) | 0.002 (0.002–0.002) |
| ALK | 0.001 (0.001–0.001) | -0.000 (-0.000–0.000) | 0.001 (0.001–0.002) | 0.002 (0.002–0.002) | 0.002 (0.001–0.002) |
| **Squamous cell carcinoma (N = 90)** | | | | | |
| Baseline AUC | 0.842 (0.755–0.923) | 0.829 (0.733–0.915) | 0.828 (0.730–0.916) | 0.833 (0.739–0.918) | 0.836 (0.742–0.916) |
| **AUC decrement** | | | | | |
| Gene mutation | −0.004 (−0.005–-0.003) | −0.006 (−0.007–-0.005) | −0.009 (−0.009–-0.008) | −0.007 (−0.008–-0.007) | −0.004 (−0.005–-0.003) |
| EGFR | −0.003 (−0.004–-0.003) | −0.004 (−0.005–-0.004) | −0.004 (−0.005–-0.004) | −0.003 (−0.004–-0.003) | −0.002 (−0.003–-0.001) |
| ALK | −0.001 (−0.002–-0.000) | −0.002 (−0.003–-0.002) | −0.005 (−0.005–-0.004) | −0.004 (−0.005–-0.004) | −0.002 (−0.003–-0.001) |

^a^EGFR, epidermal growth factor receptor

^b^ALK, anaplastic lymphoma kinase.

^c^For each feature group, we performed 100 permutations to obtain the AUC decrement distribution (baseline AUC − permuted AUC) and estimated the 95 % CI by bootstrapping these drops 1,000 times. The feature group ‘gene mutation’ ranked 5th–6th out of 9 groups (‘histology’ group was excluded as all patients had the same histology) across all five models.

^d^AUC, area under the receiver operating characteristic curve

**Supplementary Figure 1**. Discrimination performance of the five Hyperband-tuned model variants (Models A–E) on the held-out test set. (A) Receiver operating characteristic curve and (B) precision-recall curve. (A) Receiver operating characteristic curves and (B) precision-recall curves for the five candidate models evaluated on the test cohort.
AUPRC, area under the precision-recall curve; AUROC, area under the receiver operating characteristic curve.


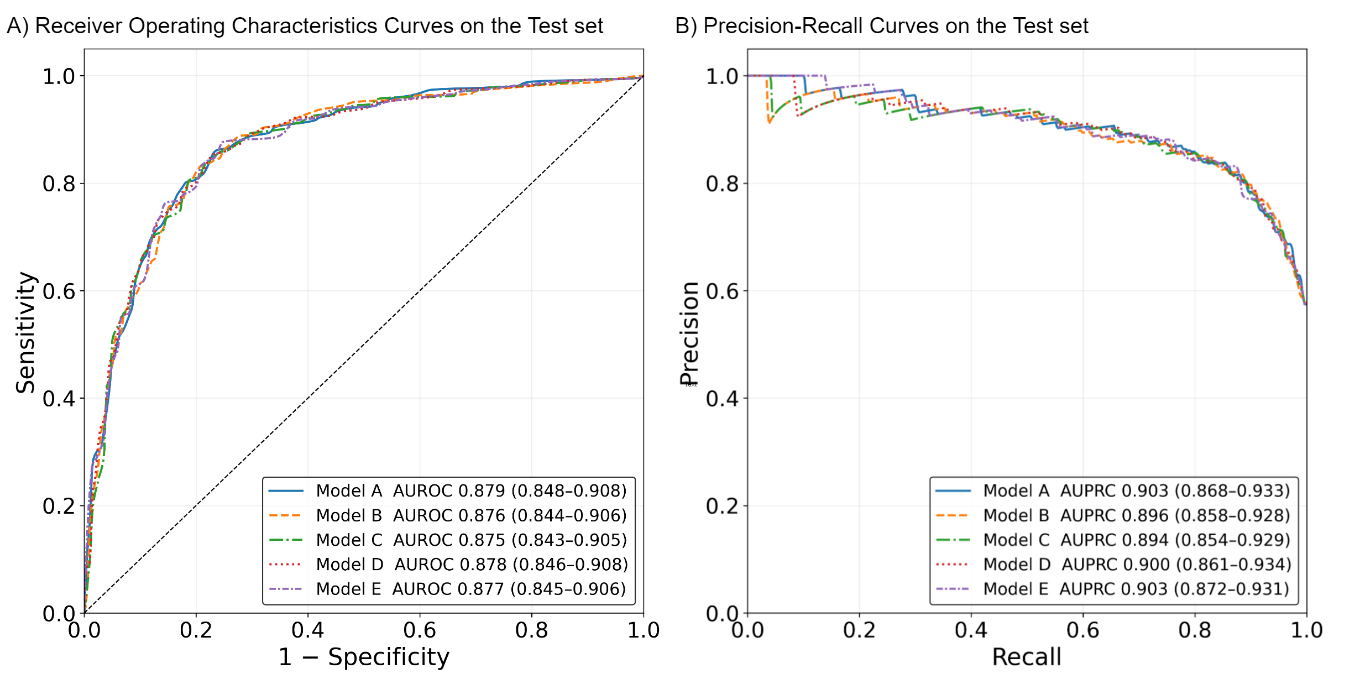


**Supplementary Figure 2.** Calibration plot of the five Hyperband-tuned model variants (Models A–E) on the test set.
**
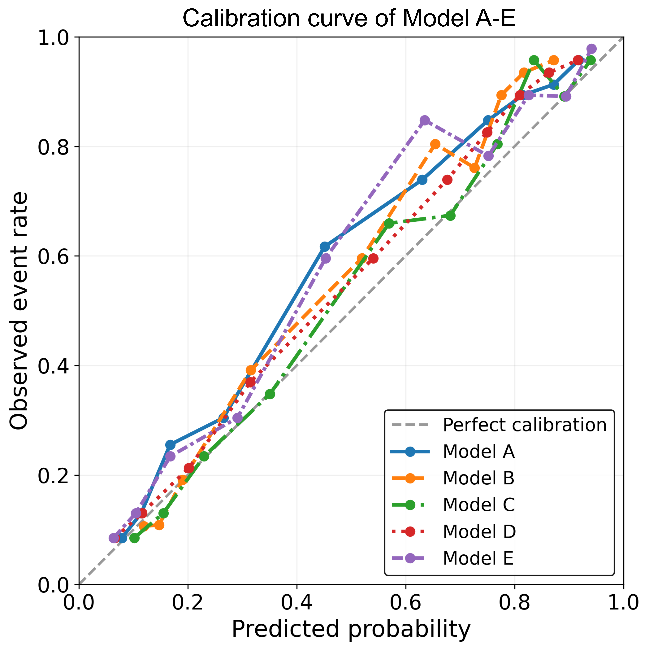
**
